# Impact of social fairness perception on sense of social security in China’s COVID-19 pandemic: the mediating role of political trust

**DOI:** 10.3389/fpsyg.2025.1525343

**Published:** 2025-07-24

**Authors:** Jianwen Wang, Jingwen Zhang

**Affiliations:** ^1^School of International Relations and Public Affairs, Shanghai International Studies University, Shanghai, China; ^2^School of Languages and Cultures, Shanghai University of Political Science and Law, Shanghai, China

**Keywords:** social fairness perceptions, sense of social security, mediating effect, political trust, COVID-19

## Abstract

**Introduction:**

Against the backdrop of the COVID-19 pandemic, emerging threats previously obscured were revealed and instilled a profound sense of insecurity across the globe. The exacerbation of unequal access to essential resources during the pandemic, particularly in rural-urban divides (e.g., healthcare infrastructure, economic relief distribution), has objectively contributed to a decline in individuals’ perceptions of social security, with rural residents facing compounded vulnerabilities. Thus, an exploration of the interplay between the variables of social fairness perception and sense of social security is warranted to provide empirical evidence and guidance for improved social governance and policy formulation in response to future social challenges.

**Methods:**

This study, grounded in the data sourced from the 2021 China Social Survey (CSS 2021), utilizes the ordinal multivariate regression model within SPSS to conduct an in-depth exploration of the intrinsic relationship between social fairness and residents’ perceptions of social security. Results: Through the application of hierarchical multivariate stepwise regression analysis, the study reveals that both opportunity fairness (β = 0.41, *p* < 0.001) and outcome fairness (β = 0.43, *p* < 0.001) significantly predicted heightened perceptions of social security. These effects remained robust after controlling for demographic variables (e.g., age, residence) and contextual factors (e.g., living environment, social welfare). Mediation analysis using bootstrapping (5,000 resamples, bias-corrected) revealed that political trust partially mediated the relationship between fairness perceptions and social security. Specifically, political trust accounted for 20.7% of the total effect of opportunity fairness (indirect effect: β = 0.10, 95% CI [0.06, 0.15]) and 27.8% of the effect of outcome fairness (indirect effect: β = 0.12, 95% CI [0.08, 0.17]). Moderated mediation analysis further indicated urban-rural disparities: urban residents exhibited significantly stronger mediation through outcome fairness (β = 0.15) compared to rural counterparts (β = 0.11).

**Discussion:**

The findings extend institutional trust theory by demonstrating that procedural equity (e.g., transparent policy implementation) reinforces governance legitimacy during crises. To mitigate disparities, policymakers should standardize urban-rural welfare systems to address unequal mediation pathways and institutionalize participatory equity audits in crisis governance frameworks. These measures operationalize the critical link between fairness perceptions and societal resilience.

## Introduction

Social security, defined as the ability of the community to preserve the essential characteristics under changing conditions and threats (Biggs, 2002), is one of the basic human needs intertwined with other essential needs such as food, clothing and housing, and a decrease in sense of security can destroy people’s sense of wellbeing and increase tension, stress, and anxiety (Chinekesh et al., 2017). The perception of social security pertains to individuals’ subjective assessments regarding the capacity of the social system to sustain effective operations and coordinated development over a specified time frame. This encompasses a comprehensive evaluation of the extent to which their legitimate rights and interests are both infringed upon and protected (Yao and Wang, 2011). Furthermore, it also reflects people’s recognition of the prevailing social security landscape, as well as their confidence in future societal developments (Guo, 2020). Therefore, comprehending the current status of social security perceptions is crucial for fostering sustainable and stable societal development.

The COVID-19 pandemic starkly exposed global vulnerabilities in this construct. The UN report *New Threats to Human Security in the Anthropocene* pointed out that people’s sense of safety and security is at a low in almost every country, including the richest countries, despite years of upward development success (Morrissey, 2022). One of the important determinants for the declining security sense is the abrupt onset of the COVID-19 pandemic, which has instilled a profound sense of fear across the globe. However, the pandemic does not affect everyone equally, and it magnifies pre-existing class differences and reveals a social gap among individuals living in the same country, even in the same city (Cardoso et al., 2020). Nielsen and Albertsen (2023) argue that social inequality permeates the circumstances in which the health risks and social threats of the pandemic arise. The disproportionate impact of the pandemic on populations worldwide is inextricably linked to the inequality in health care, incomes, access to COVID-19 vaccinations, resource distribution, etc. (Wright and Merritt, 2020; Figueroa et al., 2021; Patel et al., 2020; Stok et al., 2021). It has been found that a perception of societal injustice can exacerbate social conflicts and contradictions, indirectly affecting people’s sense of social security (Wei et al., 2014; Goda et al., 2023), therefore what should be taken into account is the alteration of perceived social fairness in the pandemic since it has highlighted the interconnectedness of various dimensions of human security and revealed social vulnerability that was previously obscured (Fallah-Aliabadi et al., 2022).

This holds true for China. Although China has made remarkable achievements in economic and social development in recent decades, the unequal distribution of resources has been a persistent social problem in China (Yu and Wang, 2016). Given the soaring income inequalities prior to the pandemic (Xie and Zhou, 2014) and its disproportionate impact on low-skilled workers, rural-to-urban migrants, and fresh graduates from university (Che et al., 2020; Wang et al., 2021), some scholars have posited that inequalities may further increase during and possibly after the pandemic (He et al., 2022). As the disparities wrought by the COVID-19 pandemic deteriorate, the sense of social security, which encompasses feelings of safety, stability, and wellbeing, has been significantly altered. This paper presents an empirical study based on the Chinese Social Survey 2021, examining the relationship between social fairness perception and the sense of social security among Chinese citizens during the COVID-19 pandemic.

## Literature review and research hypotheses

### Social fairness perception and sense of social security

Social fairness denotes the equitable allocation of societal resources across political, economic, and social domains, with public perceptions of injustice shaping subjective evaluations of equity (Yu, 2005). Research has shown that both distributive fairness and procedural fairness are positively associated with members’ psychological security (Zhang et al., 2015), and unfairness deriving from competitive economic environments is often associated with negative societal consequences (Stiglitz, 2012; Verhaeghe et al., 2015). An imbalance in perceived societal fairness can lead to the accentuation of social conflicts and contradictions, affecting social harmony and stability (Tao et al., 2015; Wei et al., 2014; Li and Qin, 2012), and populations residing in societies marked by significant social inequalities tend to exhibit diminished perceptions of social security (Yang, 2016). The unfairness in the process of social resource allocation and the unjust outcomes of distribution place vulnerable groups in a state of “relative deprivation” or even “absolute deprivation,” which is the root cause of social security incidents (Zhou, 2008).

Social fairness perception encompasses two dimensions: perceptions of opportunity fairness and perceptions of outcome fairness. Opportunity fairness pertains primarily to citizens’ beliefs in equal rights and opportunities, whereas outcome fairness relates to individuals’ perceptions of equitable access to economic benefits and social status (Jiang and Wang, 2003). Outcome fairness specifically captures individuals’ evaluations of income and wealth distribution. Individuals’ past experiences and exposure to external inequality significantly influence their subjective perception of inequality and their preferences for redistribution (Marandola and Xu, 2021). Perceptions of systemic resource exclusion—such as unequal access to pandemic relief—heighten both personal and collective relative deprivation, particularly through eroded confidence in institutional fairness (Guo, 2001), thereby triggering public dissatisfaction, resistance, and disillusionment with governmental actions, ultimately lowering their perception of social security. As the widening disparity in income, public services, healthcare, and other resources between urban and rural populations, as well as among different social strata, exacerbates this problem, marginalized groups in China are increasingly prone to feeling social injustice and heightened insecurity due to these disparities (Li and Qin, 2012). Furthermore, an individual’s sense of security is directly correlated with their ability to exert control over external circumstances. Greater control over the external environment can effectively enhance one’s perception of security (Li, 2007). Consequently, those who suffer from unfair treatment are more inclined to perceive the social environment as insecure (Zhu, 2020). These adverse responses, coupled with dissatisfaction stemming from perceived social injustice, profoundly affect residents’ sense of social security (Chen and Zhang, 2022). In light of these observations, the following hypothesis is proposed:

*H1a*: Higher perceptions of outcome fairness positively predict residents’ sense of social security.

Opportunity fairness is commonly understood to signify the provision of equitable life chances for all citizens, implying that individuals should be accountable for their efforts and choices but not for exogenous circumstances beyond their control (Roemer and Trannoy, 2016; Scanlon, 2018). This concept is considered more important than outcome fairness by many (Trautmann and van de Kuilen, 2016; Hou, 2020), as dynamically adjusting opportunities through policy levers such as eligibility thresholds or quota adjustments is seen as more feasible than redistributing monetary outcomes (Trautmann, 2023). Opportunity fairness ensures the fundamental rights and interests of every individual in society by shaping choices in the labor market, education, health, and other aspects of life. Drawing on perceived control theory (Bandura, 1997), restricted opportunities undermine individuals’ sense of agency, amplifying security risks. An unfair competitive social environment lacking equality of opportunity, such as through discriminatory educational policies or affirmative action, can erode trust in social institutions, thereby decreasing security perceptions. Such discriminatory policies undermine individuals’ fair participation and can trigger social dissatisfaction and insecurity incidents (Wang and Huo, 2019). Furthermore, achieving opportunity fairness is crucial for maintaining a fair and equitable society, ensuring that everyone has the chance to succeed based on their efforts and choices, and the absence of scientific mechanisms for the readjustment and distribution of resources exacerbates outcome disparities between different social groups, posing a challenge to social security and stability (Song and Wang, 2010). Emerging evidence underscores how pandemic-induced disparities amplified opportunity inequality, particularly in critical domains like education and healthcare access. Wang and Li (2020) found that disaster-affected people exhibited higher fairness perceptions of government relief policies and subsequent life satisfaction when social security measures were deemed satisfactory. This underscores the potential of social security satisfaction to positively reinforce the realization of social fairness. However, the mediating role of political trust in this relationship remains underexplored. From this, the following hypothesis is proposed:

*H1b*: Opportunity fairness perceptions positively predict residents’ sense of security.

### The mediating effect of political trust

Rousseau et al. (1998) conceptualize trust as “a psychological state” characterized by the willingness to accept vulnerability, grounded in positive expectations of another’s intentions or behavior. The OECD report (OCED, 2015) further specifies trust in government as “the confidence of citizens and businesses in the actions of government to do what is right and perceived as fair.” Van de Walle and Bouckaert (2003) elaborate on this, viewing trust as a direct consequence of the disparity between citizens’ expectations and their perception of the government’s actual performance. Similarly, Bélanger and Carter (2008) describe trust as “individuals’ perceptions of the integrity and ability of the service-providing agency.” Easton (1975), on the other hand, defines political trust as the likelihood that “members feel their interests will be addressed even if authorities are subject to minimal supervision or scrutiny.”

Political trust refers to residents’ evaluation and confidence in the government, political system, and public officials based on the operational performance and psychological expectations of these entities (Hetherington, 1998). A political system’s fundamental task involves determining which policy outputs are implemented and who benefits from them, as highlighted by Easton (1965) and Dahl (2000). Political institutions can exacerbate inequality through various means, including public spending, job subsidies, wage regulations, and the perpetuation of rent-seeking tactics (Salverda et al., 2009; Stiglitz, 2012). Consequently, social fairness plays a crucial role in shaping political trust (Lee, 2021), as unfairness in the protection of democratic rights, judicial and law enforcement opportunities, as well as unfair distribution of income and social welfare, can all contribute to reduced levels of political trust among residents (Zhao and Yu, 2017; Zhi et al., 2022). For instance, Eric Uslaner’s research on the relationship between income inequality and political trust reveals that as income inequality increases, levels of both social and political trust decline (Uslaner, 2002). Similarly, Zhang (2016), utilizing data from the China Family Panel Studies, demonstrates that personal experiences of injustice significantly influence individuals’ evaluations of the government. Those who have encountered unreasonable government fees, conflicts with officials, delays or denials of service, or societal injustices related to gender, household registration status, or wealth disparities tend to hold lower evaluations of the government.

Political trust-building significantly enhances social security by strengthening residents’ confidence in institutional capacity to mitigate risks (Zhao and Wei, 2013; Bernardi and Gotlib, 2022). Greater trust in politics fosters a belief among residents that the country and government possess the capability to address diverse risks and threats (Devine et al., 2023), thereby ensuring a secure social environment. Enhancing political trust can mitigate social conflicts and elevate the level of social security, further reinforcing residents’ sense of security (Bursik, 1988). Conversely, when survival and development opportunities are perceived as unfair across various aspects, residents tend to scrutinize the rationality of government policy-making and the integrity of political operations (Amirkhanyan et al., 2023). This leads to a distrust of the political system and a skepticism that their legitimate rights and interests will be protected. Consequently, their inner anxiety and uncertainty escalate, cultivating a profound sense of social insecurity, as observed by Zhang and Xu (2015) and Khodyakov (2007).

When residents possess firm confidence and positive expectations in the political system, institutions, or actors, they develop secure psychological cognitions and expectations, culminating in a heightened sense of social security, while this sense diminishes when such confidence and expectations are lacking (Xu, 2013). Therefore, a perception of social injustice can trigger a crisis of political trust, ultimately giving rise to a sense of social insecurity (Du, 2020). Based on the above analysis, this paper proposes the following research hypotheses.

*H2a*: Outcome fairness perceptions positively predict social security through political trust.
*H2b*: Opportunity fairness perceptions positively predict social security via political trust.

The mediating role of political trust may be further shaped by systemic disparities between urban and rural China. Rural populations, often excluded from equitable access to healthcare and economic relief (He et al., 2022), are more reliant on government welfare, potentially amplifying the importance of outcome fairness in fostering trust. In contrast, urban residents, with greater exposure to market-driven opportunities, may prioritize procedural fairness (e.g., transparent policy implementation) as a basis for political trust (Xu et al., 2020). Enria et al. (2021) illuminate the critical moderating role of policy transparency in repairing trust-fairness dynamics during systemic crises. This mechanism resonates with the rural-urban divergence observed in our study, suggesting that differential transparency in local governance (e.g., clarity of pandemic relief criteria) may explain why fairness perceptions translate into trust more effectively in urban contexts. These contextual differences suggest that the strength of political trust’s mediation between fairness perceptions and security may vary across rural and urban settings, though the core mediation hypotheses (*H2a, H2b*) are expected to hold universally.

### Other factors influencing social security

Besides social fairness, existing literature shows that factors such as demographic characteristics, living environment, and social order have statistically significant correlations with residents’ sense of social security. Firstly, in terms of demographic characteristics, older individuals (Rountree and Land, 1996), unmarried groups (Zhang, 2020), and rural residents (Wang, 2008) report heightened social security, with community cohesion and stability serving as key buffers against insecurity. As for characteristics such as income, education, and internet usage, existing studies report inconsistent findings (Li, 2007; Meng, 2012). Internet usage is included as a control variable to account for its potential influence on security perceptions. Prior studies suggest that internet access may expose individuals to fragmented or critical information, amplifying perceptions of societal risks and eroding both sense of security and political trust (Wang et al., 2023). Regarding the living environment, positive perceptions of living environments correlate with heightened security (Li, 2007; Wei and Ruan, 2011; Johnston, 2001). Lastly, low crime rates and civil stability strengthen security perceptions; the more disordered and uncivilized the society is, the more likely residents are to feel insecure and anxious (Markowitz et al., 2001; May and Dunaway, 2000). Beyond individual-level factors, institutional safeguards—such as robust welfare systems—further reinforce people’s confidence in coping with risks and significantly improve residents’ sense of social security (Zhu, 2010; Yang et al., 2020). Based on previous research results, this research will employ the aforementioned influencing factors as control variables in exploring the relationship between people’s perceived social fairness and their social security sense.

## Data and variables materials and methods

### Data sources and sample composition

The analytical foundation of this study derives from the 2021 China Social Survey (CSS 2021), a nationally representative probability survey administered by the Institute of Sociology, Chinese Academy of Social Sciences. Employing a stratified three-stage Probability Proportional to Size (PPS) design enhanced by Computer-Assisted Residential Sampling (CARS), the survey utilized digital mapping and randomized address selection to minimize fieldworker bias (Yang and Li, 2024). The design covered 31 provinces, with hierarchical sampling of 151 socioeconomically stratified county-level PSUs and 604 villages/neighborhood committees, yielding 10,268 validated resident interviews and 604 community questionnaires.

All analyses strictly applied CSS’s precomputed composite weight variable, formally documented in CSS methodology to inherently correct clustering effects (Zou and Li, 2022), which inherently corrects clustering effects by integrating base weights for differential PSU selection probabilities, non-response calibration via iterative raking, and post-stratification alignment with national age × gender × hukou distributions. Crucially, no *ad hoc* adjustments were performed by the research team, as the CSS’s integrated methodology automatically resolves clustering effects through its layered calibration structure. Following exclusion of cases with missing core variables, the analytical sample comprised 3,570 adults aged ≥ 18 years.

### Variable selection

#### Dependent variable: social security

The dependent variable is operationalized through the question: *How do you perceive the current overall situation regarding social security?* Given the notably low proportion of respondents identifying as *very insecure*, the responses categorized as *very insecur*e and *insecure* are aggregated into a single category labeled *insecure*. Responses are recoded into three categories: *insecure* (1), *secure* (2), and *very secure* (3).

#### Independent variable: social fairness

Social fairness is evaluated through the inquiry: *What is your perception of the fairness associated with the following dimensions of current social life?* The two most basic dimensions for the public to judge the fairness and reasonableness of resource allocation are opportunity fairness perceptions and outcome fairness perceptions (Meng, 2012). Opportunity fairness includes the content of distribution fairness, involving opportunities for people to move upward, narrowing the gap, and the possibility of catching up with others, while outcome fairness focuses on the final result of this social resource allocation. Thus, the social fairness under assessment consists of opportunity fairness encompassing elements such as the college entrance examination system, citizens’ political rights, judicial and law enforcement, and employment opportunities, as well as outcome fairness, including factors such as access to public healthcare, distribution of wealth and income, social welfare, and disparities in rights and benefits between urban and rural populations.

The final response categories are assigned numerical values as follows: *very unfair* (1), *unfair* (2), *fair* (3), and *very fair* (4), with higher numerical scores indicative of a stronger perception of social equity. Subsequently, we conduct exploratory factor analysis (EFA) on the constructs of opportunity equity and outcome equity. The findings, presented in Table 1, reveal that Bartlett’s test of sphericity for both constructs yielded *p*-values of less than 0.001. Moreover, the Kaiser-Meyer-Olkin (*KMO*) test indicates sampling adequacy for both opportunity fairness (*KMO* = 0.76) and outcome fairness (*KMO* = 0.80), indicating acceptable levels of sampling adequacy.

**TABLE 1:** Factor analysis of opportunity fairness and outcome fairness.

Social fairness	Variable	Mean	SD	Extraction
Opportunity fairness	College entrance examination system	3.20	0.75	0.47
	Political rights	3.05	0.74	0.70
	Judiciary and law enforcement	3.08	0.71	0.67
	Work and employment opportunities	2.84	0.71	0.53
Reliability	Cronbach’s α = 0.77			
Outcome fairness	Public healthcare	3.01	0.70	0.56
	Wealth and income distribution	2.68	0.79	0.64
	Social security benefits	2.88	0.76	0.70
	Urban-rural rights and benefits	2.66	0.80	0.69
Reliability	Cronbach’s α = 0.82			

SD, standard deviation, Cronbach’s α values exceed 0.70, indicating adequate reliability.

This indicates a significant correlation between the variables concerning opportunity fairness and outcome fairness, making them suitable for factor analysis. Following principal component factor analysis of the variables utilizing the maximum variance rotation method, one common factor with an eigenvalue greater than 1 is extracted from opportunity fairness and outcome fairness, resulting in cumulative contribution rates of 59.34 and 64.65%, respectively. These factors are designated as opportunity fairness and outcome fairness.

#### Mediating variable: political trust

The inquiry item *To what extent do you trust the following institutions?* is employed to assess the level of trust in central government, district and county governments, township administrations, judicial courts, and public security departments. Responses categorized as *don’t know* or marked as *missing* are excluded from the subsequent statistical analyses. The remaining valid responses are organized into four distinct categories: *complete distrust* (1), *moderately distrustful* (2), *trustworthy* (3), and *very trustful* (4), resulting in a four-level categorical variable.

Following the categorization, exploratory factor analysis of the variable political trust, as delineated in Table 2, reveals that Bartlett’s test of sphericity was significant (*p* < 0.001), and the Kaiser-Meyer-Olkin measure of sampling adequacy was 0.73. These outcomes suggest a significant interrelationship among the variables, thereby reinforcing the appropriateness of the dataset for factor analysis. Principal component analysis with varimax rotation identifies a single common factor (eigenvalue > 1) explaining 63.23% of the variance. This factor is designated as political trust.

**TABLE 2:** Factor analysis of political trust.

Variable	Mean	SD	Extraction
Central government	3.65	0.56	0.37
District and county government	3.21	0.75	0.74
Township government	3.05	0.83	0.68
Courts	3.19	0.72	0.68
Public security department	3.24	0.71	0.69
Reliability	Cronbach’s α = 0.85		

Cronbach’s α values exceed 0.70, indicating adequate reliability.

#### Control variables

In alignment with the existing literature, this study incorporates several control variables that may influence residents’ perceptions of social security. These control variables include age, marital status, internet usage, place of residence, social welfare, living environment, social appraisal, social morality, and law-abidingness.

Internet usage (0 = no, 1 = yes) and place of residence (0 = rural, 1 = urban) are operationalized as dummy variables. Marital status is collapsed into a binary dummy variable, where *never married* amalgamates responses from unmarried and cohabiting individuals (coded as 0), while *ever married* encompasses responses from individuals in first marriages, remarriages, divorces, and widowhood (coded as 1). Age is calculated based on the year of birth. Social welfare and living environment are measured using two items: *Overall, how do you assess the social security situation?* and *How satisfied are you with the living environment?* Social appraisal is determined through the question, *Overall, how do you evaluate society?* Social morality is gauged using the question: *How would you evaluate the overall moral standards of individuals in contemporary society?* Lastly, law-abidingness is assessed based on the question: *How would you evaluate the level of compliance with laws and regulations among individuals in society?* All responses are measured as continuous variables on a scale from 1 to 10.

#### Descriptive statistics and bivariate correlations

This study implements a multifaceted approach to mitigate common method bias (CMB). The China Social Survey (CSS 2021) integrated procedural safeguards to address CMB, including optimized research design, standardized data collection, statistical testing, and quality control. These measures aimed to enhance data validity by reducing method-related threats. Specifically, the survey utilized randomized question ordering and temporal separation during questionnaire design, alongside multi-source validation (e.g., cross-checking responses against administrative records) during data collection. Respondents were explicitly informed that answers were unlinked to personal identifiers, reducing social desirability bias. These structured design strategies significantly weaken systematic errors stemming from a single method variance source at the measurement level. Statistically, Harman’s single-factor test (Podsakoff et al., 2003) is conducted. An unrotated principal component analysis (PCA) identified three common factors with eigenvalues greater than 1, among which the first factor explained 36.6% of the variance, below the critical threshold of 40%. This quantitative evidence indicates that CMB does not substantially interfere with the theoretical relationships among the core variables of this study, ensuring the statistical robustness of the findings.

The descriptive statistics for the study sample (*N* = 3570) are presented in Table 3. A majority of respondents reported feeling secure (70.6%) or very secure (24.4%) regarding social security, with only 5.0% perceiving insecurity. The sample was nearly evenly distributed between rural (51.3%) and urban (48.7%) residents, suggesting balanced geographical representation. Key continuous variables—Political Trust (*M* = 0, *SD* = 1.78), Opportunity Fairness (*M* = 0, *SD* = 1.54), and Outcome Fairness (*M* = 0, *SD* = 1.61)—were standardized and exhibited substantial variability, reflecting diverse perceptions of institutional trust and equity. The comparable dispersion between Opportunity and Outcome Fairness (*SD*s = 1.54 vs. 1.61) further indicates heterogeneous evaluations of procedural and distributive justice. While the low proportion of social insecurity (5.0%) may imply overall societal stability, the urban-rural balance highlights the need to examine potential localized disparities in resource access—a focus aligned with the study’s emphasis on equity and security dynamics. The notably low incidence (5.0%) of insecure responses primarily reflects China’s unique pandemic governance context. This distribution arises from three interrelated mechanisms: First, stringent lockdown policies objectively reduced immediate health threats, temporarily elevating security perceptions. Second, collectivist cultural norms may incentivize reporting bias toward socially desirable responses (Jiang et al., 2022). Third, reliance on a single-item measurement scale obscures nuanced vulnerability patterns, particularly among marginalized groups. Consequently, while indicating robust societal resilience, this skewed distribution likely underestimates true insecurity drivers and underrepresents high-risk populations.

**TABLE 3:** Descriptive statistics of variables (*N* = 3,570).

Category	Variable level	Freq. (%)	Mean	SD	Range
Social security	Insecure	177(5.0)			
	Secure	2,523(70.6)			
	Very secure	870(24.4)			
Marriage	No	615(17.2)			
	Yes	2,955(82.8)			
Internet usage	No	883(24.7)			
	Yes	2,687(75.3)			
Residence	Rural	1,832(51.3)			
	Urban	1,738(48.7)			
Mediating/independent	Political trust		0.00	1.78	−7.07 to 2.27
	Opportunity fairness		0.00	1.54	−5.60 to 2.64
	Outcome fairness		0.00	1.61	−4.73 to 3.12
Control	Age		43.67	14.47	18.00–70.00
	Social Welfare		7.27	2.34	1.00–10.00
	Social appraisal		7.95	1.57	1.00–10.00
	Living environment		7.50	2.09	1.00–10.00
	Social morality		7.46	1.82	1.00–10.00
	Lawabidingness		7.75	1.75	1.00–10.00

SD, standard deviation; range formatted as Min–Max.

The correlation matrix (Table 4) is analyzed to assess preliminary relationships among variables central to the study’s focus: social fairness perceptions (opportunity and outcome fairness), political trust, and social security. Both opportunity fairness (*r* = 0.55, *p* < 0.01) and outcome fairness (*r* = 0.49, *p* < 0.01) exhibit strong positive correlations with political trust. These results align with institutional justice theory, suggesting that equitable resource allocation enhances citizens’ confidence in governance systems. Perceived fairness in opportunities (*r* = 0.36, *p* < 0.01) and outcomes (*r* = 0.36, *p* < 0.01) are moderately associated with social security, supporting hypotheses *H1a* and *H1b*. This underscores the role of procedural and distributive equity in fostering societal stability. Political trust correlates positively with social security (*r* = 0.34, *p* < 0.01), providing initial evidence for its mediating role (*H2a*/*H2b*). This implies that fairness perceptions may amplify security indirectly by strengthening institutional trust. Urban residency negatively correlates with social security (*r* = −0.09, *p* < 0.01), reflecting unequal access to crisis-relief resources between urban and rural populations. Welfare satisfaction (*r* = 0.27, *p* < 0.01) moderately strengthens security perceptions, emphasizing the importance of material safeguards during crises. Older individuals report slightly higher security (*r* = 0.11, *p* < 0.01), potentially due to accumulated resilience or broader social safety nets. This analysis supports the hypothesized linkages between fairness perceptions, political trust, and social security, while highlighting urban-rural and welfare-related disparities.

**TABLE 4:** Correlation matrix (*N* = 3,570).

	OF	OCF	PT	Age	SocAp	Law	Welf	Moral	SocSec
OF	1								
OCF	0.70**	1							
PT	0.55**	0.49**	1						
Age	−0.02	−0.09**	0.00	1					
SocAp	0.40**	0.40**	0.41**	0.12**	1				
Law	0.35**	0.33**	0.35**	0.14**	0.59**	1			
Welf	0.37**	0.41**	0.41**	−0.01	0.49**	0.42**	1		
Moral	0.37**	0.36**	0.37**	0.15**	0.60**	0.66**	0.44**	1	
SocSec	0.36**	0.36**	0.34**	0.11**	0.44**	0.33**	0.27**	0.32**	1
Res	−0.05**	−0.04*	−0.03	−0.18**	−0.07**	−0.08**	0.01	−0.08**	−0.09**
Marr	−0.10**	−0.08**	0.60**	0.06**	0.09**	−0.04*	0.09**	0.04*	−0.18**
NetUse	−0.03*	−0.03	−0.06**	−0.49**	−0.06**	−0.00	0.11**	0.60**	0.05**

Two-tailed test: *p < 0.05, **p < 0.01. OF, Opportunity Fairness; OCF, Outcome Fairness; PT, Political Trust; SocAp, Social Appraisal; Law, Lawabidingness; Welf, Social Welfare; Moral, Social Morality; SocSec, Social Security; Res, Residence; Marr, Marriage.

#### Model selection

Given that the social security status of residents constitutes a three-category ordinal variable, the ordered logit model is deemed appropriate for data analysis.

The regression model is:


Security=iα+α1Fairness+iα2X+iεi

Among the variables, *Security _*i*_* denotes the social security status of resident i; *Fairness _*i*_* encapsulates the concept of social fairness to be deconstructed, which includes perceived opportunity fairness and perceived outcome fairness. *X _*i*_* represents a composite set of control variables, encompassing age, marital status, internet usage, place of residence, social welfare, living environment, social appraisal, social morality, and law-abidingness. ε *_*i*_* denotes a random error term.

A fundamental assumption underpinning ordinal logistic regression is the proportional odds assumption, which posits that the relationship among each pair of outcome categories remains consistent. The results of the parallel lines test conducted in this study yield a non-significant outcome (Model 1, *p* = 0.107 > 0.05; Model 2, *p* = 0.213 > 0.05; Model 3, *p* = 0.199 > 0.05; Model 4, *p* = 0.302 > 0.05), indicating that the assumption holds validity and that the selected model is appropriate for the analysis.

### Data analysis

#### Basic regression analysis

The relative impact of predictors on social security was examined through regression models. First, we compared the coefficient of determination (*R*^2^) across nested models to assess model fit improvement. A substantial increase in this coefficient indicates that the newly introduced variables enhance the model’s explanatory power, thereby demonstrating the statistical significance of the regression model. Secondly, the *P*-value serves as an indicator of whether a significant linear relationship exists within the equation. *p* > 0.05 are interpreted as non-significant, while *p* ≤ 0.05 indicate statistically significant effects. We further examine standardized coefficients (β) to assess the relative strength and direction of each variable’s association with social security. The direction and magnitude of this influence further demonstrate the significance of the relationship between the explanatory and dependent variables.

As illustrated in Table 5, four regression models are specified. Model *M1* incorporates all control variables. Model *M2* introduces the explanatory variable of opportunity fairness. The Pseudo *R*^2^ value for *M2* increases from 0.29 to 0.33, demonstrating a significantly improved explanatory capacity for residents’ social security, and opportunity fairness shows a significant effect (*p* < 0.001), suggesting its substantive role in explaining social security. Subsequently, Model *M3* incorporates the independent variable of outcome fairness on the basis of *M1*, yielding a Pseudo *R*^2^ value of 0.33, indicating that *M3* explains additional variance compared to *M1*. Outcome fairness, with *p* < 0.001, indicates that it is also an effective explanatory variable for residents’ social security. On the basis of *M1*, both the independent variables, opportunity fairness and outcome fairness, are incorporated in *M4*. With pseudo-*R*^2^ = 0.341, it shows better fit than previous models.

**TABLE 5:** Ordinal regression of influencing factors of social security (*N* = 3,570).

Variable	Model 1	Model 2	Model 3	Model 4
Age	1.01*** (1.00–1.02)	1.01*** (1.00–1.02)	1.01*** (1.01–1.02)	1.01*** (1.01–1.02)
Social welfare	1.05*** (1.01–1.10)	1.02 (0.98–1.06)	1.00 (0.96–1.04)	1.00 (0.96–1.04)
Social appraisal	1.77*** (1.64–1.90)	1.68*** (1.55–1.81)	1.67*** (1.55–1.81)	1.66*** (1.53–1.79)
Social Morality	1.03 (0.97–1.09)	0.99 (0.93–1.05)	0.99 (0.93–1.05)	0.98 (0.92–1.04)
Lawabidingness	1.12*** (1.06–1.20)	1.10*** (1.03–1.17)	1.10*** (1.03–1.18)	1.10*** (1.03–1.17)
Living environment	1.11*** (1.07–1.16)	1.10*** (1.05–1.15)	1.10*** (1.05–1.15)	1.10*** (1.05–1.14)
Internet usage (No)	1.18 (0.96–1.44)	1.17 (0.95–1.44)	1.11 (0.90–1.37)	1.13 (0.93–1.39)
Marriage (No)	1.31 (1.05–1.63)	1.12 (0.86–1.47)	1.22 (0.94–1.58)	1.14 (0.87–1.50)
Residence (Rural)	1.28*** (1.09–1.51)	1.24*** (1.06–1.46)	1.23*** (1.04–1.45)	1.23*** (1.04–1.45)
Opportunity Fairness	–	1.41** (1.33–1.50)	–	1.21*** (1.18–1.25)
Outcome fairness	–	–	1.43*** (1.35–1.51)	1.27** (1.18–1.37)
Test of parallel lines	0.11	0.21	0.20	0.30
Pseudo R^2^	0.29	0.33	0.33	0.34

OR values rounded to two decimal places; 95% CI in parentheses (en-dash format). Pseudo R2 values indicate the proportion of variance explained by the model. Significance: ***p < 0.001, **p < 0.01.

Among control variables, age, social appraisal, living environment, law-abidingness, and rural residency significantly predicted higher social security. Older adults’ heightened security may reflect cumulative social trust, while rural residents’ reliance on community cohesion could buffer against instability (He et al., 2022). In contrast, factors such as Internet usage, marital status, and moral evaluations of society do not demonstrate a statistically significant effect on residents’ sense of security. Furthermore, the relationship between social welfare and residents’ sense of security exhibits a degree of instability; particularly notable is the observation that when the variables of opportunity fairness and outcome fairness are introduced, the impact of social welfare fails to achieve statistical significance.

Both opportunity fairness (*OR* = 1.41, *p* < 0.001) and outcome fairness (*OR* = 1.43, *p* < 0.001) strongly predicted security perceptions (supporting *H1a* and *H1b*). Marginal effects indicated that each unit increase in opportunity and outcome fairness raised the probability of higher security by 12.1% and 12.7% respectively. These findings align with institutional trust theory: when policies (e.g., pandemic relief) are perceived as procedurally fair (opportunity fairness) and distributively just (outcome fairness), citizens report greater security (Rousseau et al., 1998). The finding indicates that when individuals perceive that governmental policies, healthcare, and economic relief efforts are distributed fairly during the pandemic, they are more likely to feel secure. Conversely, perceived inequalities or favoritism in policy responses and resource allocation can exacerbate feelings of vulnerability and insecurity.

#### Mediating effect test

To investigate the intrinsic mechanisms linking social fairness (comprising opportunity fairness and outcome fairness) to social security, this study adopts a dual-path moderated mediation framework. Political trust, operationalized as a composite index of confidence in governmental institutions, serves as the mediator, while urban-rural residency (coded as 0 = rural, 1 = urban based on *hukou* status) functions as the moderator. The analysis explicitly examines how both dimensions of fairness—opportunity (e.g., equitable access to education and employment) and outcome (e.g., income and healthcare equity)—interact with residency to shape security perceptions. Using PROCESS Macro Model 7 (Hayes et al., 2017), we separately analyze the indirect effects of opportunity fairness and outcome fairness on social security through political trust, testing moderation by residency via interaction terms (e.g., Opportunity Fairness × Residency; Outcome Fairness × Residency). We estimate bias-corrected bootstrap confidence intervals (5,000 resamples, BCa method) for conditional indirect effects and moderated mediation indices. Control variables (e.g., social welfare, living environment) were included to isolate fairness-specific mechanisms. While the analysis prioritizes political trust as the mediator, the dual fairness framework acknowledges potential parallel pathways, warranting future exploration through chain mediation models or multi-group SEM to address spatial and sequential complexities.

As shown in Table 6, opportunity fairness exerts a statistically significant positive influence on political trust (β = 0.45, *p* < 0.001). This finding corroborates the institutional justice theory (Tyler, 2006) positing that equitable resource allocation mechanisms reinforce citizens’ legitimacy attribution to governmental systems. Specifically, a one-unit increase in opportunity fairness (e.g., enhanced policy transparency or educational equity) corresponds to a 0.45-unit elevation in political trust, underscoring institutional equity as a pivotal determinant of public political attitudes. Living environment positively predicted both political trust (β = 0.04, *p* < 0.01) and social safety (β = 0.02, *p* < 0.001), supporting its dual role in enhancing institutional and public security perceptions. Social appraisal strongly associated with higher political trust (β = 0.10, *p* < 0.001) and social safety (β = 0.09, *p* < 0.001), indicating cross-domain reinforcement of societal evaluations. Internet usage negatively linked to political trust (β = -0.15, *p* = 0.03) and social safety (β = -0.05, p = 0.02), suggesting systemic erosion of trust and security through digital engagement.

**TABLE 6:** Regression results for political trust and social security perception.

Variable	Political trust model		Social security model	
	Coefficient (SE)	95% CI	Coefficient (SE)	95% CI
**Main predictors**				
Opportunity fairness	0.45*** (0.02)	[0.40, 0.49]	0.05*** (0.01)	[0.04, 0.06]
Political trust	–	–	0.03*** (0.01)	[0.02, 0.04]
**Moderation effects**				
Residence (Urban = 1)	0.04 (0.05)	[−0.05, 0.14]	–	–
Opportunity fairness × residence	0.05 (0.03)	[−0.01, 0.12]	–	–
**Controls**				
Living environment	0.04** (0.01)	[0.01, 0.07]	0.02*** (0.00)	[0.01, 0.02]
Social welfare	0.12*** (0.01)	[0.09, 0.14]	−0.00 (0.00)	[−0.01, 0.01]
Social appraisal	0.10*** (0.02)	[0.06, 0.14]	0.09*** (0.01)	[0.07, 0.10]
Social morality	0.06** (0.02)	[0.02, 0.09]	−0.00 (0.01)	[−0.01, 0.01]
Lawabidingness	0.04 (0.02)	[−0.00, 0.07]	0.02** (0.01)	[0.00, 0.03]
Marriage	−0.25** (0.08)	[−0.40, −0.09]	−0.00 (0.02)	[−0.05, 0.04]
Internet usage	−0.15*x (0.06)	[−0.28, −0.03]	−0.05* (0.02)	[−0.08, 0.01]
Age	0.00 (0.00)	[−0.00, 0.00]	0.00** (0.00)	[0.00, 0.00]

Political trust model *R*^2^ = 0.378, *F*(11, 3,558) = 196.69, *p* < 0.001. Social Security Model *R*^2^ = 0.255, *F*(10, 3,559) = 121.74, *p* < 0.001. Significance: ***p < 0.001, **p < 0.01, *p < 0.05.

Political trust serves as a significant partial mediator in the relationship between opportunity fairness and social security perception. Opportunity fairness exhibits a direct effect on social security perception (β = 0.05, *p* < 0.001), independent of political trust, potentially mediated by non-psychological mechanisms such as economic safeguards or legal-institutional optimizations. The indirect effect of opportunity fairness through political trust remains significant across rural (β = 0.01) and urban (β = 0.02) subgroups, accounting for 20.7% of the total effect. This supports the institutional trust-social stability linkage model, wherein political trust functions as a conduit translating institutional equity into societal stability.

As for the moderating effect of residence, urban-rural residence does not significantly moderate the “opportunity fairness → political trust” pathway (β = 0.05, *p* = 0.079; Δ*R*^2^ = 0.0005; Table 7). The index of moderated mediation (0.002, 95% CI [−0.001, 0.004]) straddles zero, indicating insufficient heterogeneity between urban and rural populations in trust formation mechanisms. This non-significance may reflect that China’s urban-rural integration initiatives (e.g., equalized access to basic public services) may have reduced disparities in institutional perceptions. Alternatively, the binary urban-rural classification could mask localized disparities, such as between urban villages and suburban areas, necessitating finer-grained spatial stratification.

**TABLE 7:** Moderated mediation analysis of opportunity fairness (x) on social security (Y) through political trust (M) by residence (W)

Effect	β	Estimate (SE)	95% CI	*R* ^2^
**Direct effect**				
Opportunity fairness (X→Y)	0.051***	0.006	[0.040, 0.063]	0.255
**Indirect effects**				
-Rural (W = 0, X→M→Y)	0.013**	0.003	[0.008, 0.019]	0.378
-Urban (W = 1, X→M→Y)	0.015**	0.003	[0.009, 0.021]	
**Moderated effect**				
Interaction term	0.05	0.031	[−0.01, 0.12]	
Moderated mediation index	0.002	0.001	[−0.001, 0.004]	Δ*R*^2^ = 0.0005

Total variance explained (R^2^): 25.5% for Y (Social Security), 37.8% for M (Political Trust). Significance: ***p < 0.001, **p < 0.01.

The findings on the mediating role of political trust align with Rousseau et al.’s (1998) conceptualization of trust as a psychological state rooted in positive expectations of others’ intentions or behaviors. During the COVID-19 pandemic, perceived opportunity fairness, such as equitable access to education and employment, enhanced citizens’ confidence in the government’s commitment to procedural justice. This confidence, as our data show (β = 0.636, *p* < 0.001 for opportunity fairness → political trust), reflects Rousseau’s emphasis on trust as a dynamic process shaped by institutional performance and perceived equity. Specifically, the government’s efforts to ensure fair resource allocation (e.g., vaccine distribution policies) amplified political trust, which in turn bolstered social security perceptions.

**FIGURE 1:**
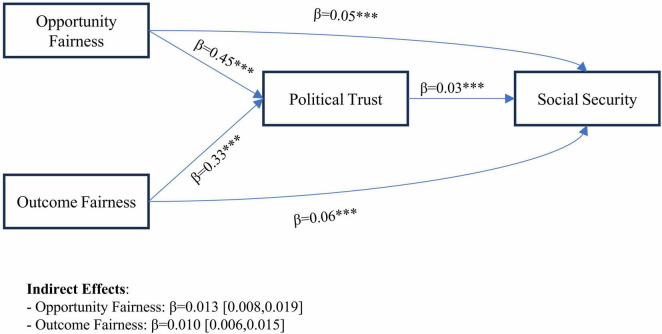
Mediation model. Significance: ***p < 0.001.

Similarly, the findings from another mediation model analysis (Table 8) reveal that outcome fairness significantly enhances residents’ sense of social security through both direct and indirect pathways, with political trust serving as a critical mediator. A one-unit increase in perceived outcome fairness (e.g., equitable distribution of income or healthcare resources) elevates political trust by 0.33 units (β = 0.33, *p* < 0.001). In turn, political trust amplifies social security perceptions by 0.03 units (β = 0.03, *p* < 0.001), as shown in Figure 1. This mediation mechanism also aligns with Rousseau et al.’s (1998) theory of trust as a psychological state rooted in institutional performance. During crises such as the COVID-19 pandemic, equitable outcomes in resource allocation (e.g., economic relief distribution) likely reinforced citizens’ confidence in governmental efficacy, thereby reducing anxiety and fostering a resilient sense of security. Living Environment, Social Appraise, Lawabidingness, and Age demonstrated consistent positive associations in both models. Living Environment has significant coefficients in the Political Trust Model (β = 0.05, *p* < 0.001) and the Social Security Model (β = 0.02, *p* < 0.001). Age showed incremental positive effects in both models (Political Trust: β = 0.01, *p* < 0.05; Social Security: β = 0.00, *p* < 0.001).

**TABLE 8:** Regression results for political trust and social security perception.

Variable	Political trust model		Social security model	
	Coefficient (SE)	95% CI	Coefficient (SE)	95% CI
**Main predictors**				
Outcome fairness	0.33*** (0.02)	[0.28, 0.37]	0.06*** (0.01)	[0.05, 0.07]
Political trust	–	–	0.03*** (0.01)	[0.02, 0.04]
**Moderation effects**				
Residence	0.03 (0.05)	[−0.07, 0.13]	–	–
Outcome fairness × residence	0.08**(0.03)	[0.02, 0.14]	–	–
**Controls**				
Living environment	0.05***(0,01)	[0.02, 0.07]	0.02*** (0.00)	[0.01, 0.02]
Social welfare	0.11***(0.01)	[0.09, 0.14]	−0.01 (0.00)	[−0.01, 0.00]
Social appraise	0.12***(0.02)	[0.08, 0.16]	0.09*** (0.01)	[0.07, 0.10]
Social morality	0.07***(0.02)	[0.03, 0.10]	0.00 (0.01)	[−0.02, 0.01]
Lawabidingness	0.06**(0.02)	[0.02, 0.09]	0.02** (0.01)	[0.01, 0.03]
Marriage	−0.39*** (0.08)	[−0.55, −0.23]	−0.02 (0.02)	[−0.06, 0.03]
Internet usage	−0.10 (0.07)	[−0.23, 0.03]	−0.04 (0.02)	[−0.07, 0.00]
Age	0.01* (0.00)	[0.00, 0.01]	0.00*** (0.00)	[0.00, 0.01]

Political trust model: *R*^2^ = 0.33, *F*(11, 3,558) = 159.83, *p* < 0.001. Social Security Model: *R*^2^ = 0.26, *F*(10, 3,559) = 125.92, *p* < 0.001. Significance levels: ***p < 0.001, **p < 0.01, *p < 0.05.

**FIGURE 2:**
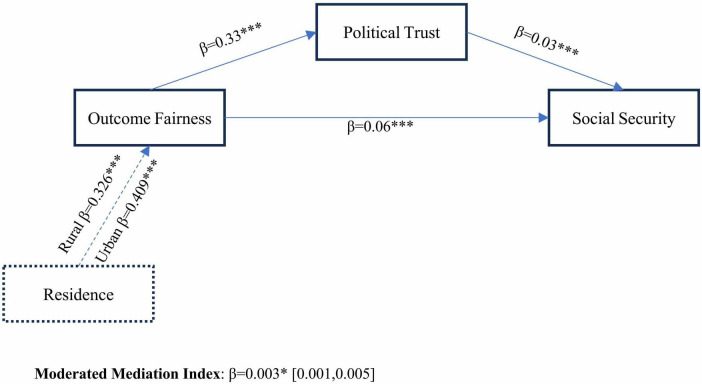
Moderated mediation model. Significance: *p < 0.05, ***p < 0.001.

The total indirect effect of outcome fairness through political trust accounts for 15.3% of the total effect, highlighting political trust as a pivotal conduit translating distributive equity into societal stability (Table 9). The residual direct effect suggests that complementary mechanisms, such as legal safeguards or economic policies (e.g., unemployment benefits), independently bolster security perceptions. Urban-rural residency significantly moderates the relationship between outcome fairness and political trust, as evidenced by a statistically significant interaction effect (β = 0.08, *p* = 0.007; Δ*R*^2^ = 0.0014). Conditional effects analysis reveals divergent patterns across residential groups. As shown in Figure 2, for the rural residents, a one-unit increase in perceived outcome fairness enhances political trust by 0.326 units (β = 0.326, *p* < 0.001, 95% CI [0.284, 0.369]), while for the urban residents, the effect intensifies to 0.409 units (β = 0.409, *p* < 0.001, 95% CI [0.360, 0.459]), indicating that urban populations derive substantially greater trust from equitable outcome distributions.

**TABLE 9:** Mediation analysis of outcome fairness (X) on social security (Y) through political trust (M) by residence (W).

Effect	β	Estimate (SE)	95% CI	*R* ^2^
**Direct effect**				
Outcome fairness (X→Y)	0.057***	0.006	[0.046, 0.068]	0.261
**Indirect effects**				
- Rural (W = 0, X→M→Y)	0.010***	0.002	[0.006, 0.015]	0.331
- Urban (W = 1, X→M→Y)	0.013***	0.003	[0.008, 0.018]	
**Moderation metrics**				
Interaction term	0.083**	0.031	[0.022, 0.144]	Δ*R*^2^ = 0.001
Moderated mediation index	0.003*	0.001	[0.001, 0.005]	

Total variance explained (R^2^): 26.1% for Y (Social Security), 33.1% for M (Political Trust). Significance: ***p < 0.001, **p < 0.01, *p < 0.05.

This moderating effect extends to the mediation pathway linking outcome fairness to social security. The conditional indirect effects demonstrate systematic heterogeneity. The index of moderated mediation (β = 0.003, 95% CI [0.001, 0.005]) confirms that urban-rural disparities significantly alter the strength of the fairness-trust-security linkage. Urban residents’ heightened sensitivity to outcome fairness may reflect their greater exposure to institutional processes (e.g., transparent policy implementation) and market-driven opportunities, which amplify expectations of distributive equity (Xu et al., 2020). Conversely, rural populations, despite relying more heavily on visible equity (e.g., welfare distribution), exhibit marginally weaker indirect effects, likely due to systemic resource gaps and delayed access to crisis-relief measures (He et al., 2022).

These findings underscore the necessity of geographically differentiated governance. Urban policies should prioritize institutional transparency to align with residents’ engagement in procedural accountability, while rural interventions must address tangible outcome disparities (e.g., healthcare subsidies, agricultural support) to bridge trust deficits. The persistence of moderated mediation effects, even at a modest magnitude, highlights the critical role of contextual factors in shaping fairness perceptions during crises.

Consequently, both *H2a* and *H2b* are empirically supported. The finding aligns with the previous empirical research on the point that people’s opportunity fairness perceptions can promote their sense of security and political trust (Zheng and Zhao, 2020), but is contrary to studies suggesting outcome fairness does not predict social security via political trust (Wang and Li, 2023; Arneson, 2018). The rising emphasis on outcome fairness may reflect China’s accelerated marketization, which prioritizes measurable economic outcomes over procedural equity (Xu et al., 2020). China’s pandemic policies—including strict quarantines, lockdowns, and mobility restrictions—may have further shaped these perceptions. These policies have led to a sudden and significant disruption of economic activities, resulting in massive unemployment, widespread wage cuts, and a sharp rise in household hardship (He et al., 2022). Consequently, the rising dependency on the government’s distribution of resources may heighten sensitivity to outcome fairness (e.g., relief equity), while diminishing attention to opportunity fairness (e.g., long-term mobility).

#### Robustness tests through regression analysis with alternative dependent variable

To mitigate potential biases arising from skewed security perceptions, we replace the single-item scale with an 8-dimensional security index, redistributing responses to ensure no category fall below 10%. Additionally, bootstrapped mediation analysis (*N* = 5,000) is employed to minimize skew-induced error and enhance confidence in the results. Following these adjustments, preliminary regression analysis redefined the dependent variable while holding independent and control variables constant. Specifically, social security is reassessed using eight items under the prompt, *How do you evaluate the level of security in the following aspects of current society?* These questions include personal and family property security, personal safety, traffic safety, medical safety, food safety, workplace safety, personal information and privacy security, and environmental safety. *Unknown* and *missing* responses are excluded, valid responses are coded into four categories: *very insecure* (1), *relatively insecure* (2), *relatively secure* (3), and *very secure* (4). The linear regression assumptions are met (normality: Shapiro-Wilk *p* = 0.15; homoscedasticity: Breusch-Pagan *p* = 0.12). Using stepwise regression (*p* < 0.05 for entry), we derive Model 5, which explained 32.6% of the variance (adjusted *R*^2^ = 0.326, *p* < 0.001). Predictors show no multicollinearity (VIF < 5) or autocorrelation (Durbin-Watson = 2.009).

The data presented in Table 10 suggests that both opportunity fairness (*p* < 0.001, 95% CI [0.34, 0.52]) and outcome fairness (*p* < 0.001, 95% CI [0.38, 0.56]) significantly predict security perceptions. Standardized coefficients indicate that outcome fairness (β = 0.20) has a slightly stronger effect than opportunity fairness (β = 0.18). These results robustly confirm that opportunity and outcome fairness are key predictors of social security, underscoring the role of institutional equity in fostering societal stability.

**TABLE 10:** Robustness check with substitution of dependent variables (*N* = 3,570).

Variables	B	SE	β	*p*	95% CI
Constant	18.61	0.38	–	<0.001	[17.86, 19.36]
Social appraisal	0.63	0.04	0.26	< 0.001	[0.55, 0.71]
Outcome fairness	0.47	0.046	0.12	< 0.001	[0.38, 0.56]
Opportunity fairness	0.43	0.05	0.18	< 0.001	[0.34, 0.52]
Internet usage	−0.38	0.14	−0.04	0.008	[−0.65, −0.10]
Living environment	0.14	0.03	0.08	< 0.001	[0.08, 0.19]
Residence (Urban = 1)	−0.33	0.11	−0.05	0.002	[−0.54, −0.12]
Age	0.01	0.00	0.05	0.003	[0.00, 0.02]

Adjusted *R*^2^ = 0.326 (32.6% variance explained); Durbin-Watson = 2.009.

To further validate the mediation model, we redefine the dependent variable and test it using PROCESS Model 4 (Hayes et al., 2017). It is found that perceived opportunity fairness (β = 0.64, *t* = 39.48, *p* < 0.001) has a statistically significant positive predictive influence on political trust, which subsequently influenced social security perceptions. Perceived opportunity fairness (β = 0.76, *t* = 17.91, *p* < 0.001) and political trust (β = 0.50, *t* = 13.63, *p* < 0.001) strongly predicted social security perceptions. Perceived outcome fairness also significantly predicted political trust (β = 0.54, *t* = 33.55, *p* < 0.001). Both perceived outcome fairness (β = 0.75, *t* = 19.43, *p* < 0.001) and political trust (β = 0.54, *t* = 15.24, *p* < 0.001) demonstrate significant positive predictive relationships with social security perceptions. The findings presented in Table 11 confirm that political trust partially mediates the influence of both opportunity and outcome fairness on security perceptions, reinforcing the role of institutional trust in crisis governance.

**TABLE 11:** Robustness check of mediating effect of political trust.

Social fairness	Effect	SE	LLCI	ULCI	% of total
**Opportunity fairness**					
Total effect	1.08	0.04	1.01	1.16	–
Direct effect	0.76	0.04	0.68	0.85	70.39%
Indirect effect	0.32	0.03	0.26	0.38	29.61%
**Outcome fairness**					
Total effect	1.04	0.04	0.98	1.11	–
Direct effect	0.75	0.04	0.68	0.85	72.20%
Indirect effect	0.29	0.00	0.24	0.34	27.80%

## Conclusion and implications

This study examines how perceptions of social fairness, both in opportunities and outcomes, shape residents’ sense of security during crises, with political trust acting as a critical mediator. Drawing on nationally representative data from the 2021 China Social Survey (CSS 2021), our empirical analysis demonstrates that opportunity fairness and outcome fairness exert significant positive effects on social security perceptions. Political trust partially mediates these relationships, accounting for 20.7% and 27.8% of the total effects, respectively. These findings underscore that fairness is not merely a normative ideal but a structural determinant of societal resilience, particularly evident during the COVID-19 pandemic, where systemic inequities in resource distribution amplified vulnerabilities among marginalized groups (He et al., 2022). The weaker mediation effect of political trust among rural residents aligns with global patterns of regional trust disparities (Dellmuth, 2023). Rural populations often face compounded vulnerabilities: limited access to crisis-relief information and delayed policy implementation exacerbate perceptions of procedural injustice, even when material outcomes (e.g., subsidy amounts) are nominally equitable.

The mediation role of political trust aligns with institutional theory, which posits that equitable governance fosters public confidence in crisis management (Rousseau et al., 1998). Specifically, procedural fairness in policy implementation (e.g., transparent resource allocation) enhances trust in governmental efficacy, thereby attenuating societal tensions and promoting compliance with public health measures, a dynamic observed cross-nationally during the pandemic (Blair et al., 2022; Oksanen et al., 2020). However, urban-rural disparities persist: urban residents exhibited stronger mediation effects from outcome fairness, likely due to greater exposure to institutional accountability mechanisms, whereas rural populations remained disproportionately affected by material inequities (Chen and Li, 2022). Notably, the study identifies demographic heterogeneities. Older adults and individuals with positive evaluations of their living environment reported heightened security, reflecting cumulative socioeconomic stability and adaptive capacities. Conversely, systemic inequalities in education, healthcare, and welfare access exacerbate relative deprivation among disadvantaged groups, eroding their capacity to mitigate risks (Mezzina et al., 2022; Zhou, 2008). These disparities, if unaddressed, may perpetuate cycles of insecurity and social deviance, as evidenced by rising crime rates in regions with pronounced fairness deficits (Wang and Huo, 2019).

Our findings advance the literature on crisis governance by delineating the mechanisms linking multidimensional fairness to societal stability. While prior studies emphasize material security (Stiglitz, 2012), this research highlights the psychosocial pathways through which perceived equity reinforces institutional legitimacy. Policymakers must prioritize equity audits in crisis frameworks, targeting rural-urban resource gaps and institutionalizing participatory feedback mechanisms to align policies with community needs. The empirical findings highlight that institutionalizing fairness in opportunity structures and outcome distribution is pivotal for enhancing political trust and social security. To operationalize these insights, we propose three core evidence-based strategies. First, implement digital platforms to systematically capture public perceptions of fairness, particularly among marginalized groups. This addresses the study’s mediation results showing that procedural fairness in resource allocation significantly strengthens political trust. Secondly, integrate community-led evaluations into relief frameworks to align policies with localized needs, mitigating urban-rural disparities in healthcare access (He et al., 2022). This responds to the moderated mediation effects, where urban populations exhibited heightened sensitivity to outcome fairness, underscoring the need for context-specific procedural justice. Thirdly, prioritize funding for regions with historically low fairness perceptions to counteract adaptive capacity erosion. Regression models revealed rural residents’ weaker indirect security gains despite reliance on visible equity, necessitating investments in rural digital infrastructure and healthcare subsidies to bridge systemic gaps (Chen and Li, 2022; Wang et al., 2024). These strategies operationalize the critical nexus between fairness perceptions, political trust, and societal resilience. By embedding equity-driven mechanisms into governance, policymakers can transform crisis vulnerabilities into opportunities for sustainable security, fostering long-term stability amid systemic challenges.

### Limitations and future research

This study has several limitations that warrant consideration. First, although political trust emerged as a significant mediator, the socio-psychological mechanisms linking fairness to security remain incompletely mapped. Future research should explore parallel or sequential mediation pathways, such as the interplay of social support and institutional efficacy, to account for the complexity of societal resilience. Second, the Chinese context, marked by centralized crisis governance and rapid urbanization, limits generalizability. Cross-national comparative studies could clarify whether the observed mediation mechanisms are universal or culturally contingent. Integrating digital trace data (e.g., social media sentiment on fairness) with traditional surveys may further enhance the ecological validity of security perception research. By addressing these gaps, scholars can advance a more nuanced understanding of how institutional equity shapes societal stability in an era of compounding crises. Third, the low prevalence of social insecurity in our sample (5.0%) may constrain the generalizability of findings to high-risk populations. This distributional skew could obscure nuanced vulnerability dynamics among marginalized groups experiencing acute security deficits. To address this limitation, future studies should implement stratified sampling targeting high-risk cohorts (e.g., rural-to-urban migrants, informal workers) to capture heterogeneity in fairness-trust-security pathways. Additionally, employing validated multi-item measurement tools would improve sensitivity in capturing nuanced differences in security perceptions.

## Data Availability

The original contributions presented in the study are included in the article/supplementary material, further inquiries can be directed to the corresponding author.
